# Supplementary material for: The Association between ABCG2 421C>A (rs2231142) Polymorphism and Rosuvastatin Pharmacokinetics: A Systematic Review and Meta-Analysis
Source: Pharmaceutics. 2022 Feb 24;14(3):501. doi: 10.3390/pharmaceutics14030501 (PMC8954661; doi:10.3390/pharmaceutics14030501)
Supplement: Supplementary file 1 [file pharmaceutics-14-00501-s001.zip › pharmaceutics-1582781-supplementary.pdf]

pharmaceutics

MDPI

The Association between *ABCG2* 421C>A (rs2231142) Polymorphism and Rosuvastatin Pharmacokinetics: A Systematic Review and Meta-Analysis

Yubin Song †, Hee-Hyun Lim †, Jeong Yee, Ha-Young Yoon and Hye-Sun Gwak *

College of Pharmacy and Graduate School of Pharmaceutical Sciences, Ewha Womans University, Seoul 03760, Korea; 1564069@ewhain.net (Y.S.); dlagmlgus000@ewhain.net (H.-H.L.); jjjhello1@naver.com (J.Y.); hayoungdymphnayoon@gmail.com (H.-Y.Y.).

* Correspondence: hsgwak@ewha.ac.kr; Tel.: +82-2-3277-4376

† These authors contributed equally to this work.

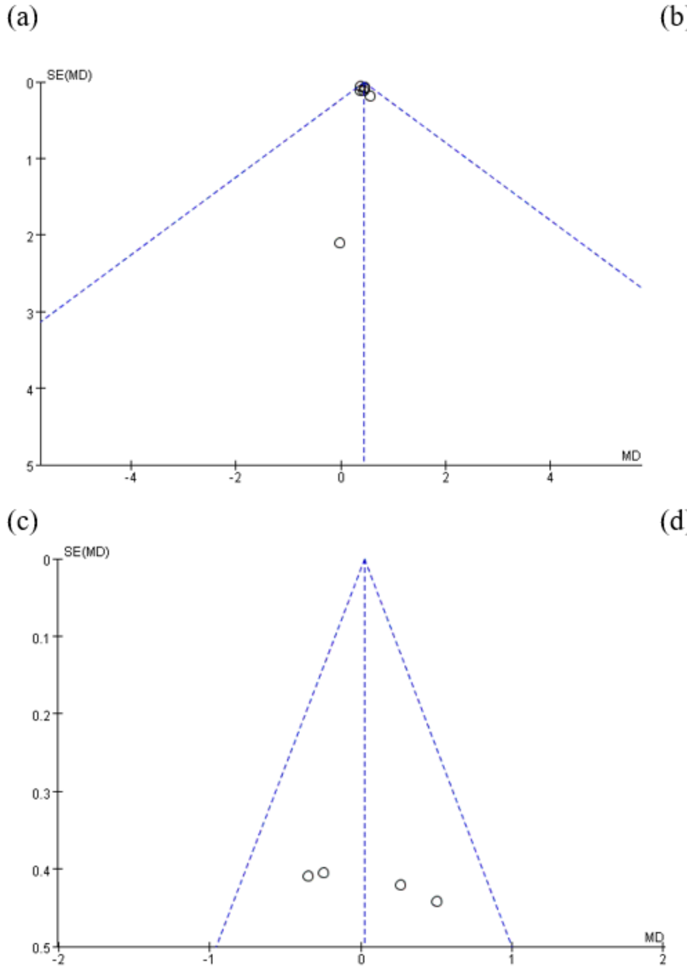

Figure S1. Funnel plot of effect of *ABCG2* 421C>A on rosuvastatin; (**a**) $AUC_{0-\infty}$ (ng·h/ml), (**b**) C_{max} (ng/ml), (**c**) t_{max} (h), (**d**) half-life (h)

Table S1. $AUC_{0-\infty}$ (ng·h/ml) and C_{max} (ng/ml) of rosuvastatin between *ABCG2* 421 A allele carriers and GG genotype carriers

Studies	Dose (mg)	Type of mean	$AUC_{0-\infty}$ (ng·h/mL)				C_{max} (ng/mL)			
			ABCG2 421 CA/AA		*ABCG2* 421 CC		*ABCG2* 421 CA/AA		*ABCG2* 421 CC	
			Mean (SD)	N	Mean (SD)	N	Mean (SD)	N	Mean (SD)	N
Birmingham 2015a	20	G	179.0 (1.5)	33	114.0 (1.5)	54	18.8 (1.5)	33	11.1 (1.6)	54
Birmingham 2015b	20	G	216.7 (1.5)	60	148.8 (1.5)	78	23.4 (1.6)	60	15.9 (1.6)	78
Huguet 2016	10	G	26.9 (15.5)	2	27.5 (20.3)	14	N/A	N/A	N/A	N/A
Keskitalo 2009	20	A	95.2 (32.1)	16	62.3 (22.7)	16	10.0 (4.2)	16	7.1 (3.1)	16
Kim 2017	20	G	N/A	N/A	N/A	N/A	19.9 (10.7)	9	16.2 (8.4)	4
Liu 2016	20	A	239.4 (140.6)	34	153.8 (66.5)	27	25.3 (13.9)	34	16.2 (8.4)	27
Wan 2015	10	A	177.5 (58.0)	34	112.7 (38.2)	28	16.5 (7.8)	34	10.9 (3.6)	28
Zhang 2006	20	A	62.2 (23.5)	7	34.9 (11.9)	7	9.9 (5.4)	7	5.1 (2.4)	7

A: arithmetic mean; AUC: area under the curve; G: geometric mean; N/A: not available; SD: standard deviation.

Table S2. Sensitivity analysis of rosuvastatin $AUC_{0-\infty}$ (ng·h/ml)

Excluded study	lnGM	95% CI	I^2
Birmingham 2015a	0.42	0.33 to 0.51	0
Birmingham 2015b	0.45	0.35 to 0.54	0
Huguet 2016	0.43	0.35 to 0.50	0
Keskitalo 2009	0.42	0.34 to 0.51	0
Kim 2017	0.43	0.35 to 0.50	0
Liu 2016	0.43	0.35 to 0.51	0
Wan 2015	0.42	0.33 to 0.50	0
Zhang 2006	0.42	0.34 to 0.50	0
TOTAL	0.43	0.35 to 0.50	0

GM: geometric mean difference; CI: confidence interval

Table S3. Sensitivity analysis of rosuvastatin C_{max} (ng/ml)

**Excluded study**	**lnGM**	**95% CI**	**I^2**
Birmingham 2015a	0.39	0.29 to 0.49	0
Birmingham 2015b	0.44	0.33 to 0.55	0
Keskitalo 2009	0.43	0.33 to 0.52	0
Kim 2017	0.42	0.33 to 0.51	0
Liu 2016	0.42	0.32 to 0.52	0
Wan 2015	0.44	0.34 to 0.54	0
Zhang 2006	0.41	0.32 to 0.50	0
TOTAL	0.42	0.33 to 0.51	0

GM: geometric mean difference; CI: confidence interval

Table S4. Sensitivity analysis of rosuvastatin t_{max} (h)

Excluded study	AMD	95% CI	I^2
Keskitalo 2009	0.12	-0.36 to 0.59	8
Liu 2016	-0.12	-0.59 to 0.34	0
Wan 2015	-0.06	-0.53 to 0.41	14
Zhang 2006	0.15	-0.33 to 0.63	0
TOTAL	0.02	-0.39 to 0.43	0

AMD: arithmetic mean difference; CI: confidence interval

Table S5. Sensitivity analysis of rosuvastatin half-life (h)

Excluded study	AMD	95% CI	I^2
Keskitalo 2009	0.11	-0.91 to 1.13	1
Liu 2016	0.21	-0.78 to 1.20	0
Wan 2015	-0.57	-2.03 to 0.89	0
Zhang 2006	0.13	-0.97 to 1.23	3
TOTAL	0.05	-0.91 to 1.01	0

AMD: arithmetic mean difference; CI: confidence interval
